# Income assurances are a crucial factor in determining public compliance with self-isolation regulations during the COVID-19 outbreak – cohort study in Israel

**DOI:** 10.1186/s13584-020-00418-w

**Published:** 2020-10-20

**Authors:** Moran Bodas, Kobi Peleg

**Affiliations:** 1 Israel National Center for Trauma & Emergency Medicine Research, The Gertner Institute for Epidemiology and Health Policy Research, Sheba Medical Center, Tel Hashomer, 5265601 Ramat-Gan, Israel; 2 The Department of Emergency Management & Disaster Medicine, School of Public Health, Sackler Faculty of Medicine, Tel-Aviv University, Tel Aviv, Israel

**Keywords:** COVID-19, Compensation, Compliance, Self-quarantine, Attitudes, Cohort

## Abstract

**Background:**

The outbreak of a new Coronavirus disease (COVID-19) poses dramatic challenges to public health authorities worldwide. One measure put in place to contain the spread of the disease is self-quarantine of individuals who may have been exposed to the disease. While officials expect the public to comply with such regulation, studies suggest that a major obstacle to compliance for self-quarantine is concern over loss of income or employment due to the prolonged absence from work.

**Methods:**

A cohort study of the adult population of Israel was conducted in two time points during the COVID-19 outbreak, the last week of February and the third week of March 2020, in order to assess public attitudes. In particular, public compliance rates to self-quarantine with and without State-sponsored compensation for lost wages were assessed.

**Results:**

The results suggest that public attitudes changed as the threat increased, making people more compliant with regulations. In February 2020, compliance rate for self-quarantine dropped from 94% to less than 57% when monetary compensation for lost wages was removed; however, in March 2020 this drop became more moderate (from 96 to 71%). The multivariate logistic regression revealed that older, non-Jewish, worried over COVID-19, and trusting the Ministry of Health were more likely than their counterparts to comply with self-isolation, even when monetary compensation was not assumed.

**Conclusions:**

Despite the effects of threat on people’s obedience with regulations, this study demonstrates that providing people with assurances about their livelihood during absence from work remains an important component in compliance with public health regulations.

## Background

A novel Severe Acute Respiratory Syndrome (SARS) coronavirus originated in December 2019 from Wuhan, Hubei, China. The disease caused by this virus (a.k.a. COVID-19) has since spread throughout the globe causing major disruption to ordinary life [23, 40]. As of 19 April 2020, a total confirmed cases of 2,241,359 cases of COVID-19 were confirmed globally, of which 152,551 died [39].

Health authorities struggle to contain the spread of COVID-19 by implementing a variety of measures designed to reduce the rate of infection. These include social distancing, hygiene, use of gloves and masks in public areas, etc. [14]. One of the main measures employed by health authorities in the effort to combat the spread of infectious diseases is self-quarantine [13, 18]. Several studies have demonstrated the applicability of self-quarantine specifically in reducing COVID-19 morbidity [12, 16, 20] or increasing public perception of its efficacy in doing so (e.g., [10]).

Accordingly, individuals who are thought to have been exposed to COVID-19 are instructed to place themselves under household self-quarantine for a minimum of 14 days. Prior research suggest that during disease outbreaks, the public views self-quarantine favorably and is willing to comply with such regulation [5]. Consequently, officials expect the public to comply with such regulation; however, studies suggest that a major obstacle to compliance for self-quarantine is concern over loss of income or employment due to the prolonged absence from work [4, 33].

Public cooperation with regulations such as self-isolation are vital for these measures to be effective in slowing down the spread of diseases. Non-compliance with self-isolation can lead to increased morbidity and mortality and set back national efforts to contain the outbreak. Such behavior is dependent on numerous factors, including public fear [17] or concern [30], possible penalties for breaching regulations [36], perceived cost of protective measures ([11], attitude toward solitude [37], information overload [11, 19], working conditions, particularly being able to work from home [25], whether the self-isolation is voluntary or mandatory [34], and general preparedness [10].

Therefore, it is imperative to assess public attitudes toward compliance with health regulation posed during the COVID-19 outbreak, in particular self-quarantine. In an earlier publication, we reported the results of a survey performed in the last week of February 2020 in Israel [6]. At that time, only five cases of COVID-19 had been confirmed in Israel, and more than 5000 Israelis put in self-quarantine at their homes. The results of that study suggested a major effect of monetary compensation for lost wages on the intent of people to comply with self-quarantine regulation. The compliance rate for self-quarantine dropped from 94% to less than 57% when monetary compensation for lost wages was removed. Since then, additional studies have demonstrated the impact of monetary compensation on compliance with self-isolation [3].

In fact, the State of Israel offered several monetary incentives to prompt individuals to comply with self-isolation and other health regulations, including state-sponsored unemployment benefits for individuals who lost their source of income during the COVID-19 outbreak valid until June 2021, as well as financial compensation for businesses and self-employed individuals, depending on the level of loss they experienced.^1^ In addition, the government issued single time grants for each citizen made payable through the National Insurance Institute [29].

The purpose of the current study was to assess the same public attitudes toward the COVID-19 outbreak that were assessed in February 2020 at a later stage of the outbreak (March 2020). The comparison between the two time points allows the assessment of change in public attitudes as the threat of COVID-19 increased and progressed.

## Methods

This cohort study was conducted in Israel during February–March 2020. Randomized samples of the adult population of Israel were engaged in a study to assess public attitudes concerning the outbreak of COVID-19. Responses were collected through the *iPanel* online polling service. Since 2006, the *iPanel* provides an online platform for a wide variety of information collection services, including polls and public opinion surveys. It adheres to the stringent standards of the world association for market, social, and opinion researchers (ESOMAR). In addition, the *iPanel* was evaluated by the Applied Statistical Laboratory of the Hebrew University in Jerusalem and was found to be highly accurate in assessing internet-based samples of the adult Jewish population in Israel [31].

A representative sample of the adult population of the State of Israel was assessed on two time points: last week of February and third week of March 2020. On each time point, a different sample was assessed, making the two sample mutually exclusive. The final sample for February included 563 respondents and the final sample for March 2020 included 511 respondents, representing response rates of about 25% each, similar to other studies that utilized the *iPanel* (e.g., [2, 7, 8, 24]). Table 1 summarizes the sociodemographic breakdown of the studied samples. At the time responses were collected, the following circumstances were evident: In the last week of February 2020 a total number of five cases of COVID-19 were confirmed in Israel and more than 5000 Israelis were required to self-quarantine at home. In March 2020 there were 677 confirmed COVID-19 cases (of which about a dozen were serious), and approximately 60,000 Israelis were required to self-quarantine at home.

**Table 1:** Socio-demographic distribution (n (%)) of studied samples (Feb. and Mar. 2020)^a^

Variable	Feb. 2020 ***N*** = 563	Mar. 2020 ***N*** = 511
**Gender**		
Female	284 (50.4%)	262 (51.3%)
Male	279 (49.6%)	249 (48.7%)
**Age**		
Average ± SD	39.57 ± 14.09	39.28 ± 14.24
18–35	258 (45.8%)	239 (46.8%)
36–55	213 (37.8%)	195 (38.2%)
56–70	86 (16.4%)	77 (15.1%)
**Religion**		
Jewish	456 (81.0%)	407 (79.6%)
Other^b^	107 (19.0%)	104 (20.4%)
**Religiosity**^**c**^		
Secular	290 (63.6%)	267 (65.6%)
Traditional	58 (12.7%)	52 (12.8%)
Religious	59 (12.9%)	51 (12.5%)
Ultra-Orthodox	49 (10.8%)	37 (9.1%)
**Place of residence**		
Haifa & North	199 (35.3%)	182 (35.6%)
Tel-Aviv & Center	153 (27.2%)	143 (28.0%)
South & Coastline Plain	120 (21.3%)	102 (20.0%)
Greater Jerusalem	49 (8.7%)	46 (9.0%)
HaSharon Region	42 (7.5%)	38 (7.4%)
**Birth place**		
Israel	487 (86.4%)	464 (90.8%)
Outside Israel	76 (13.6%)	47 (9.2%)
**Family status**		
Coupled	414 (73.5%)	359 (70.3%)
Single	149 (26.5%)	152 (29.7%)
**Children**		
Yes	382 (67.9%)	304 (59.5%)
└ < 18 years	320 (83.8%)	261 (85.9%)
No	181 (32.1%)	207 (40.5%)
**Family size**		
Average ± SD	3.94 ± 1.78	3.94 ± 1.78
1–2 members	137 (24.3%)	137 (24.3%)
3–5 members	337 (59.9%)	337 (59.9%)
6+ members	89 (15.8%)	89 (15.8%)
**Education**		
< K-12	95 (16.9%)	84 (16.6%)
K-12 diploma	110 (19.5%)	113 (22.1%)
Vocational	140 (24.9%)	111 (21.7%)
Bachelor’s degree	166 (29.5%)	146 (28.6%)
Master’s or above	52 (9.2%)	57 (11.2%)
**Income**		
Below average	230 (40.8%)	221 (43.3%)
Average	117 (20.8%)	98 (19.3%)
Above average	162 (28.8%)	130 (25.4%)
Missing	54 (9.6%)	61 (12.0%)
**Employment**		
Part/fulltime employed	371 (65.9%)	332 (65.0%)
Student	71 (12.6%)	71 (13.9%)
Unemployed	42 (7.5%)	37 (7.2%)
Self-employed	40 (7.1%)	34 (6.6%)
Retired	29 (5.2%)	33 (6.5%)
Missing	10 (1.8%)	4 (0.8%)
**Self-quarantine**^**d**^		
Were	–	44 (8.6%)
Currently are	–	52 (10.2%)

^a^ Samples are mutually exclusive (respondents in the first round were excluded from participating in the second round)

^b^ Includes: Muslims, Christians, and Druze

^c^ Relevant to the Jewish portion of the sample only

^d^ Relevant only to the March 2020 sample

The main tool used in the study was a questionnaire designed specifically for the purposes of this study. The tool was validated in the February round (see [6]). The questionnaire was comprised of six items assessing public attitudes toward the COVID-19 outbreak, including: news consumption (1 item), personal concern (1 item), public panic (2 items), and attitudes toward public health regulations (2 items). In addition, the questionnaire included three items assessing compliance with public health regulations on a nominal scale (Yes/No/Maybe/Do not know). The first two items assessed compliance with self-quarantine using the following text: “Assuming you were requested by a medical official to stay in self-quarantine and assuming the state will [not] compensate you for lost wages, will you stay in self-quarantine?”. The negative form of the question (using the word not) was asked after the positive form. The third item assessed willingness to report individuals violating self-quarantine by asking, “If you were asked to report to the Ministry of Health individuals violating self-quarantine decrees designed to protect public health, would you report them?” On the March 2020 round, the questionnaire also asked respondents to indicate whether they were or currently are in self-quarantine (2 items).

Statistical analysis was conducted using SPSS (ver. 25). The analysis included both descriptive and analytical methods. Chi-square test was used to evaluate difference in proportions of variable between groups. Independent samples t-Test was used to compare means between the two time points of assessment. A multivariate logistic regression was used to predict intent to comply with self-isolation in the absence of monetary compensation. The analyses was performed twice: first comparing “yes” responses to the primary outcome of compliance against “other” responses (“no”, “maybe”, and “don’t know” grouped together), and then excluding the “maybe” and “don’t know” answers to compared between “yes” and “no” responses. Only variables found to be associated with the dependent variable in the univariate analysis were introduced into the analysis. In addition, the effect of the sampling period was included in the analysis to explore differences between February and March samples. Regression performed in Enter mode and 95% Confidence Intervals were calculated. In all statistical analyses performed, a *p*-value of 0.05 or less was determined as statistically significant.

## Results

### Descriptive and univariate analysis

**Fig. 1:**
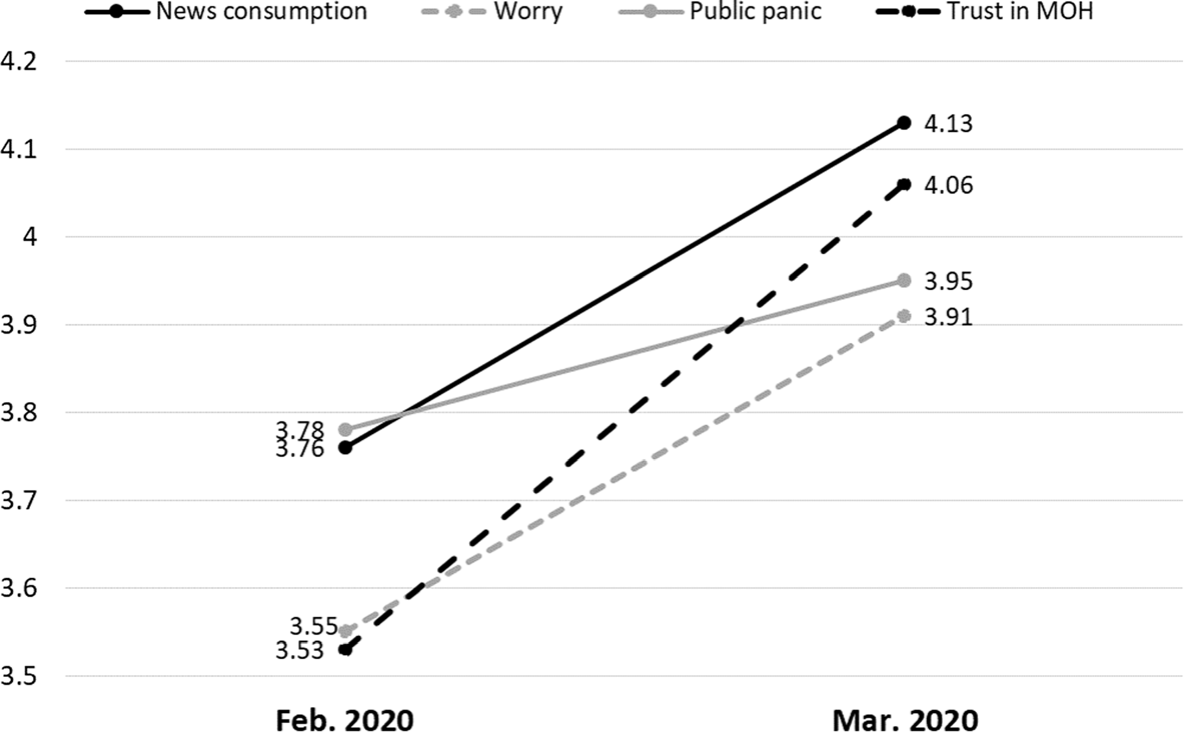
Change in mean score of attitudes toward the COVID-19 outbreak between the two rounds (*N* = 563 and *N* = 511 in February & March 2020, respectively). Mean score is computed from a range of 1 (“Not at all”) to a maximal score of 5 (“very much”). All changes are statistically significant at the *p* < .001 level. The items presented refer to the extent to which a respondent reported: (a) watching the news (“News consumption”), (b) being worried about COVID-19 (“Worry”), (c) an elevated sense of public panic (“Public panic”), and (d) trust in the regulations published by the Ministry of Health (“Trust in MOH”)

Respondents were asked to provide their attitudes toward the COVID-19 outbreak. Table 2 and Fig. 1 provide the complete results of this section. The results suggest that a majority of the Israeli public monitors the situation by consuming news reports. More than 60% in February and 77% in March reported monitoring the news “a lot” or “very much”.

**Table 2:** Distribution of responders’ attitudes (the top two options from a 5-point Likert scale) toward COVID-19 outbreak issues in the two rounds

	% of top options		% change
	February 2020 (***N*** = 563)	March 2020 (***N*** = 511)	
To what extent do you follow the news reports about the COVID-19 outbreak?	61.4%	76.5%	+ 15.1%
To what extent are you worried by the COVID-19 outbreak?	49.9%	66.6%	+ 16.7%
To what extent do you think the public is reacting in panic to the COVID-19 outbreak?	62.8%	67.3%	+ 4.5%
To what extent do you think the media is contributing to public concerns over COVID-19?	80.8%	79.8%	−1.0%
To what extent do you trust the public health instruction of the Ministry of Health during the COVID-19 outbreak?	52.8%	74.5%	+ 21.7%
To what extent do you think taking criminal action against individuals violating quarantine decree will increase compliance with MOH instructions?	69.2%	78.7%	+ 9.5%

When asked in February if they are worried about the COVID-19 outbreak, almost half of the respondents replied “a lot” or “very much”, as oppose to about 16% replying “not at all” or “a little”. By March 2020, 66% replied they were worried about the outbreak “a lot” or “very much”, and only 8% replied “not at all” or “a little”. Most respondents (63% in Feb. and 67% in Mar. 2020) believe there is panic in the public. About 80% in both rounds ascribe this panic to the media coverage of the outbreak.

When asked to what extent they trust the public health instructions issued by the Ministry of Health (MOH) during the COVID-19 outbreak, about 53% reported a high level of trust, ~ 32% a moderate level, and ~ 15% reported a low level of trust in February 2020. The level of trust in the MOH rose in the second round (March 2020) with 74% reporting a high level of trust, ~ 22% moderate, and only ~ 4% low level. Subsequently, respondents were asked whether they believe that taking criminal action against individuals breaching self-quarantine would increase compliance with MOH instructions. Almost 70 and 79% had favorable views of this proposal in February and March 2020, respectively.

Most of the variables concerning news consumption, worry, panic perception, and trust in the MOH were correlated (see Table 3). In particular, trust in the MOH is positively correlated with news consumption reported by the respondent (R(1047) = 0.219, *p* < .001) and the level of worry form the outbreak he or she reports (R(1047) = 0.505, *p* < .001).

**Table 3:** Spearman correlations (R) between COVID-19 attitudinal components among both samples (Feb. 2020 & Mar. 2020) (*N* = 1074)

	1	2	3	4
1. News consumption				
2. Worry	0.505^***^			
3. Public panic perception	0.099^**^	0.140^***^		
4. Media contribution to public panic	0.113^***^	0.086^*^	0.345^***^	
5. Trust in the Ministry of Health	0.219^***^	0.197^***^	0.069	0.028

^***^ *p*-value <.001 level ^**^ *p*-value = .001 ^*^p-value <.01 level

**Fig. 2:**
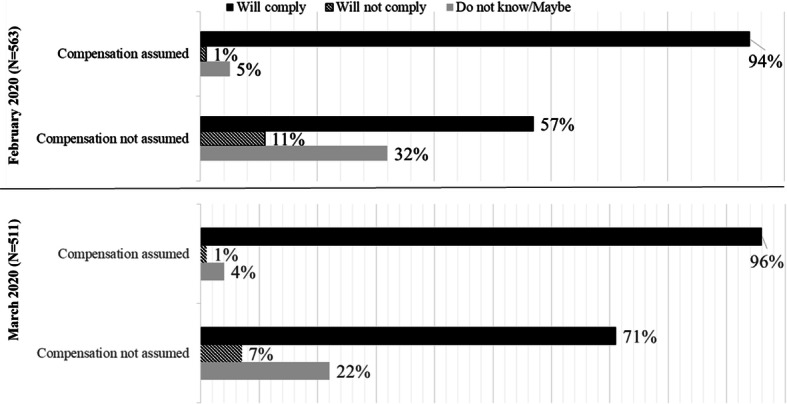
Distribution of expected rates of compliance with self-quarantine during the COVID-19 outbreak in Israel, according to whether monetary compensation for lost wages is assumed or not

The results pertaining to compliance with MOG regulation were also statistically significant. Respondents were asked to report their intent to comply with self-quarantine under two circumstances. When State-sponsored compensation for lost wages was assumed and when such compensation was removed. In February 2020, 94% of respondents indicated they would comply with a two-week self-quarantine instructed by a medical official if compensated for lost wages. Less than 1 % (0.7%) replied that they would not comply. However, when monetary compensation was removed, the compliance rate dropped to less than 57%, and 60 respondents (~ 11%) changed their response to “No”. In March 2020, the levels of compliance rose. With monetary compensation assumed, 95.5% replied they would comply with a two-week self-quarantine instructed by a medical official as oppose to 1% who would refuse. When compensation was removed, 71.4% reported they would still comply with the self-quarantine, whereas ~ 7% replied they would refuse. See Fig. 2.

When samples from February and March were pooled together to assess how employment status affects compliance with regulations, the results suggest that employed individuals are more likely than unemployed to disobey self-isolation regulations in the absence of monetary compensation for lost wages (37% versus 20%, respectively; χ^2^ = 9.227, df = 1, *p* = .002).

Taking into consideration the proportion of the groups, and in an effort to characterize individuals prone to non-compliance, the socio-demographic breakdown of respondents replying “No” or “Maybe/did not decide” were compared to those of the total sample. The analysis reveals that there are no statistical differences between the non-compliance group and the total sample (data not shown). Self-employed individuals were more prone to refuse self-quarantine measures when compensation was removed, but this finding was not statistically significant (*p* = 0.052). When compensation is removed, the group of undecided respondents (“Maybe/did not decide”) is over-representative of individuals earning higher than average income (*p* = 0.02). No other differences were observed for other socio-demographic variables between this group and the total sample (data not shown).

Respondents were asked whether they would report a person in breach of a self-quarantine decree if asked to do so by the health authorities. Most respondents (58% & 67% in February and March 2020, respectively) replied that they would report such a person to the health authorities, while 7% & 4% (respectively) would refuse to report. The remainder (35% & 29%, respectively) were unsure whether they would or would not comply with this request.

Lastly, univariate analysis was conducted to establish association between different studied factors and the intent to comply with self-isolation even in the absence of monetary compensation. To this end, the responses to this question were re-categorized into two categories: “yes” for those who indicated their intent to comply with self-isolation without compensation and “other” for all other response (“no”, “maybe”, and “do not know”). The univariate analysis suggests that gender, place of birth, and religion are associated with the intent to comply with self-isolation. More females (67.2%) than males (60.2%) (χ^2^ = 5.675, df = 1, *p* = 0.017), more Israeli-born (65.2%) than those born elsewhere (52.8%) (χ^2^ = 7.190, df = 1, *p* = 0.007), and more non-Jews (78.2%) than Jews (60.3%) (χ^2^ = 23.632, df = 1, *p* < 0.001) reported they would comply with self-isolation without compensation.

In addition, age, news consumption, worry over COVID-19, and trust in the Ministry of Health were found to be associated with the intent to comply with self-isolation. Respondents who indicated their intent to comply were older (40.46 ± 14.46) than those reporting other answers (37.64 ± 13.44), according to independent t-test (t = − 3.15, df = 855.69, *p* = 0.001). Compared to those who provided other answers, respondents who indicated their intent to comply scored higher on news consumption (4.05 ± 0.95 vs. 3.73 ± 1.00; t = − 5.148, df = 770.88, *p* < .001), worry over COVID-19 (3.88 ± 1.00 vs. 3.44 ± 1.06; t = − 6.658, df = 772.97, *p* < .001), and trust in the Ministry of Health’s regulations (3.91 ± 0.99 vs. 3.55 ± 0.98; t = − 5.702, df = 1072, *p* < .001).

### Multivariate analysis

In order to predict the intent to comply with self-isolation regulation in the absence of monetary compensation, a multivariate logistic regression was performed. Only variables found to be associated with the dependent variable in the univariate analysis were entered into the analysis. The multivariate analysis was performed both unadjusted and adjusted. The results of the regression analysis suggests that when adjusted to other explored variables, older, non-Jewish respondents who are worried about COVID-19 and trust the MOH are more likely than their respective counterparts to comply with self-isolation even when compensation is not assumed (Table 4).

**Table 4:** Adjusted and unadjusted odds-ratio of intent to comply with self-isolation without compensation for lost wages according to correlated threat perception and socio-demographic variables (*N* = 1074)

Variable^**a**^	Unadjusted		Adjusted	
	OR	95% CI	OR	95% CI
Round (Feb vs. March)	1.898	(1.472, 2.448)^+++^	1.544	(1.174, 2.032)^++^
Age	1.014	(1.005, 1.024)^++^	1.019	(1.010, 1.029)^+++^
Gender	0.739	(0.575, 0.948)^+^	0.838	(0.641, 1.096)
Place of birth	1.671	(1.145, 2.440)^++^	1.408	(0.944, 2.100)
Religion	0.423	(0.297, 0.602)^+++^	0.478	(0.327, 0.699)^+++^
News consumption	1.396	(1.228, 1.587)^+++^	1.071	(0.918, 1.250)
Worry over COVID-19	1.505	(1.330, 1.702)^+++^	1.260	(1.093, 1.474)^++^
Trust in MOH	1.428	(1.259, 1.620)^+++^	1.260	(1.100, 1.444)^++^

*OR* Odds Ratio, *CI* Confidence Interval, *MOH* Ministry of Health

^a^ Only variables found to correlate with the respective dependent variable are shown. The categorization of the independent variables are as follows: Round: February 2020–1, March 2020–2; Age: continuous; Gender: Female – 0, Male – 1; Place of birth: 0 – outside Israel, 1 – Israel; Religion: 0 – Non-Jewish, 1 – Jewish; News consumption, Worry over COVID-19, and Trust in MOH – ordinal from 1 (“not at all”) to 5 (“very much”)

^+^ *p* < 0.05 ^++^ *p* < 0.01 ^+++^ *p* < 0.001

Of note, this analysis grouped together individuals who responded “no”, “maybe” and “don’t know” to compare against individual who responded “yes” to compliance with regulations. When the same regression analysis was performed excluding “maybe” and “don’t know” response, i.e. comparing “yes” and “no” responses only, the predictors of willingness to comply with self-isolation in the absence of monetary compensation were age (B = 0.971, 95%CI: 0.954,0.989, *p* = .001), being born outside Israel (B = 0.377, 95%CI: 0.208,0.683, *p* = .001), and having trust in the health regulations (B = 0.594, 95%CI: 0.477,0.739, *p* < .001).

## Discussion

The results of this study demonstrate that as the threat of COVID-19 materialized and became to perceive as a greater risk to one’s health, so did the public attitudes changed. As the threat became more serious, participants in the studies reported elevated consumption rates of news reports, probably in an effort to stay up-to-date with current outbreak data and recommendations for self-protection. Similar findings were reported for other populations [26, 28, 32, 35]. In addition, this finding resonates similar findings reported for the Israeli population concerning a different threat, namely armed conflicts [9]. Moreover, the increased perception of the threat severity also contributed to elevated levels of worry reported by the participants in March compared to February 2020. In parallel, participants in the studies assessed that public panic is increasing as the outbreak progresses and continued to believe that media coverage of the outbreak is to blame for much of this panic.

An interesting finding from this cohort study is that as the threat perceived to be more serious and as worry and panic levels rise, the more people seem to trust the Ministry of Health, who is in charge of managing the outbreak and provide with instructions to the population. Presumably, when faced with threat, people look up to sources of credible information to get instruction on how to safeguard themselves and their loved ones. In turn, this can lead to a more favorable attitude toward that source of information, in this case, the Ministry of Health, as well as to a tendency to comply with its regulation, even in the absence of monetary compensation.

In fact, the multivariate logistic analysis highlight that having trust in the Ministry of Health is a predictor of intent to comply with self-isolation regulation, even when monetary compensation is not assumed. This is a crucial finding of this study. It stressed the importance in maintaining public trust throughout the outbreak in order to mitigate adverse consequences of the disease outbreak and to harness the public to comply with public health regulations. Similar findings were reported by other studies, including in Saudi Arabia [1] and Singapore [38].

Nevertheless, an important finding of this study is that even under the circumstances of elevated worry and greater trust in the MOH that characterized the survey carried out in March, the economic consideration continued to play a major part in determining people’s compliance with the regulations (albeit to a lesser extent than in February). Despite the moderating effect of the elevated threat perception, the results demonstrate that without monetary compensation for lost wages, some participants still indicated that they will disobey self-isolation if mandated. According to Coughlin [15], providing people with assurances about their household income during times of absence from workplace is an important component in achieving public compliance with health regulations. The results of this study demonstrate that this is true also in the case of COVID-19. Without reassurances for income, people may resort to self-help and adopt behaviors that are maladaptive in the context of public health.

This seems to be particularly true as the COVID-19 epidemic continues. The more that people get “used to” the disease and the more it take a toll on their daily lives and livelihood, the more they seem to disobey regulations,and engage inantisocial risk taking behavior [21]. Indecisive action by governments seems to aggravate this phenomenon [22]. Under such circumstances, the importance of maintaining public trust, through ensuring individuals’ economic stability, is heightened. This should come as no surprise, as Maslow’s hierarchy of needs places safety and security needs, such as employment, as second only to physiological ones [27]. Failing to address the economic implications for individuals of disease outbreaks may contribute to increased morbidity and mortality. Decision makers should be advised to take the economic implications of any disease outbreak into consideration from the get-go, and provide economic stimulations to facilitate public compliance with health regulations.

### Limitations

This study has several limitations. First, the attitudes assessed in this study reflect two time points in an ongoing health crisis. They are likely to change as the disease progresses. Second, this study utilized an internet-based methodology for response collection. While this allowed for a rapid turnover of responses on a wide geographical distribution and resulted with a representative sample of the adult population of Israel, the conclusions must be limited to people with high computer skills. Third, respondents were asked to provide assessment of their intentions, rather than reporting actual behavior, which may lead to reporting biases; moreover, the extent of such a bias could vary across population sub-groups. Lastly, the results cannot be further generalized beyond the Israeli public. Additional studies in other countries are called for to expand the database to other cultures and contexts.

## Conclusions

Public perception of the COVID-19 outbreak change over time to reflect fluctuations in the perception of the severity of the risk. This study demonstrates that as the disease progressed, Israelis responded with increased levels of worry and heightened perception of public panic. In parallel, Israelis were also more likely to view the Ministry of Health more favorably as the threat perception increased in severity and were more trusting the Ministry’s instruction. Nonetheless, this study highlights the importance of the economic considerations in managing public behavior during a disease outbreak. Continuous earning is a crucial factor in determining public compliance with public health regulations, in particular self-isolation. In the absence of monetary compensation, some people will resort to self-help, jeopardizing the integrity of the public health measures put in place to contain the spread of the disease. This phenomenon is moderated with the increase of threat perception, but is not completely eliminated by it. Decision makers are advised to put economic consideration is priority, as early as the outbreak begins.

## Data Availability

The datasets used and/or analysed during the current study are available from the corresponding author on reasonable request.

## Abbreviations

CDC: Centers for Disease Control and Prevention
COVID-19: Corona Virus Disease 2019
ESOMAR: European Society for Opinion and Marketing Research
MOH: Ministry of Health
SARS: Severe Acute Respiratory Syndrome
WHO: World Health Organization

## Footnotes

1 Compensation for part or full time employees who were put on non-paid leave included unemployment benefits up to 80% of original salary. Compensation for self-employed individuals included government grants in three stages covering 60–70% of original monthly income according to various eligibility criteria. In addition, each citizen in Israel was awarded a one-time grant, usually in the sum of 750 NIS per person, including children up to the fifth child.
